# Controlled Synthesis and Absorption Mechanism Study of FCI@UFC Absorbents

**DOI:** 10.3390/ma18051017

**Published:** 2025-02-25

**Authors:** Wenfei Yang, Zhan Gao, Yong Zhang, Hao Shi, Andong Wang, Weijie Fan

**Affiliations:** 1 Qingdao Campus, Naval Aviation University, Qingdao 266071, China; yangwf_dlut@163.com (W.Y.);; 2 School of Mechanical and Electronic Engineering, Shandong University of Science and Technology, Qingdao 266041, China

**Keywords:** microwave absorption, flake carbonyl iron powder, nitrogen-doped porous carbon, reflection loss, corrosion resistance

## Abstract

Flaky carbonyl iron (FCI) powder is a typical absorbing material with excellent magnetic loss performance. However, its single absorption mechanism, narrow effective absorption bandwidth (RL < −10 dB), poor corrosion resistance, and high density restrict the application of FCI in marine environments. In this study, carbonized urea-formaldehyde resin (UFC)-coated flaky carbonyl iron (FCI@UFC) composites were prepared by in situ polymerization and pyrolysis. Various characterization techniques were employed to investigate the phase structure, microstructure, absorption performance, and corrosion resistance of FCI@UFC. The results showed that FCI@UFC effectively combined the magnetic loss of FCI and the dielectric loss of the porous carbon layer, achieving a wider effective absorption band (EAB) with a smaller thickness. When the simulated absorption layer thickness was 1.2 mm, the EAB ranged from 10.32 to 18 GHz, which demonstrated excellent microwave absorption performance. Additionally, the porous carbon coating slowed down the direct reaction between the corrosive medium and FCI, and the porous structure could also accommodate electrolytes, maintaining a stable electrochemical environment. As a result, FCI@UFC exhibited a higher corrosion potential (−0.472 V) and a lower corrosion current (1.45 × 10^−7^ A/cm^2^), indicating good corrosion resistance. This work provides new insights for the preparation of composite materials with excellent microwave absorption and corrosion resistance in practical applications.

## 1. Introduction

The rapid development of modern electro-optical and electromagnetic detection technologies brings about great convenience in both military and civil use, but it may also pose challenges to human health, electronic devices, and military defense [1,2,3]. As a result, electromagnetic wave-absorbing technology for resisting electromagnetic radiation and radar detection has attracted significant attention. An excellent microwave absorber must exhibit properties such as light weight, low density, thin coating thickness, anti-corrosion properties, and strong absorption [4]. It is well known that magnetic materials have great potential to be outstanding microwave absorbers on account of their magnetic and dielectric loss [5].

Among the reported magnetic functional materials, flaky carbonyl iron (FCI) materials are one of the most commonly used absorptive materials with strong absorption and easy acquisition. Meanwhile, the thickness of the FCI is less than the skin depth of carbonyl iron material, which is beneficial for reducing the skin effect. Its anisotropy also helps to exceed the Snoek limit, thereby reducing the attenuation trend of FCI permeability at high frequencies and broadening its effective absorption band (EAB) [6]. However, FCI is susceptible to oxidation, exhibits poor weather resistance, and has a relatively singular electromagnetic wave loss mechanism that primarily relies on magnetic response for energy loss [7]. These shortcomings limit the further application of FCI [8].

To address the above issues, researchers commonly employ methods such as doping, forming composites, or nanosizing to modify FCI materials [9]. Among these methods, preparing core-shell structures through coating is an effective and feasible approach. The shell isolates FCI from oxygen and corrosive media while also allowing for the adjustment of the electromagnetic parameters of the iron powder, thereby enhancing the comprehensive performance of FCI. The survey found that the coating primarily consists of oxides, conductive polymers, and carbon-based materials [10,11,12,13]. For example, Zhou et al. [14] used a chemical bath deposition method to obtain SiO_2_-coated FCI composites. Madina A et al. [15] prepared carbonyl iron powder@polyaniline composite powders with core-shell structures, which improved the anti-oxidation and microwave absorption performance of the iron powder. However, the intricate synthesis process of oxides and conductive polymers, coupled with the suboptimal dielectric loss performance after coating, imposes limitations on the engineering application of FCI. Carbon-based absorbing material is a suitable candidate material, with weak magnetism and excellent dielectric properties, complementing the ultra-strong magnetic and relatively weak electric properties of FCI [16]. In this study, FCI was initially coated on urea-formaldehyde resin (UF) to obtain FCI@UF as a precursor, followed by high-temperature carbonization under a nitrogen atmosphere to fabricate FCI@UFC composites. The nitrogen doping enhanced the electronic structure and conductivity of the carbon material, while also increasing the active sites for charge storage and transfer. The formation of a porous carbon structure significantly increased the material’s specific surface area, thereby augmenting its electromagnetic wave absorption capability. Furthermore, this method also contributed to improving the material’s weather resistance, making it more suitable for applications under various environmental conditions.

Herein, this study aims to investigate the impact of nitrogen-doped porous carbon layers on the wave absorption and weather resistance of carbonyl iron powder as an absorber material. The electromagnetic parameters in the 2.0–18 GHz frequency range were tested using a coaxial ring, and the electrochemical data of materials were obtained using a three-electrode system in an electrochemical workstation. The results indicated that the FCI@UFC composite exhibited the best microwave absorption performance and resistance to electrochemical corrosion. It was found that for FCI@UFC, at a simulated absorber layer thickness of 1.2 mm, a wide absorption bandwidth of 7.68 GHz (10.32–18 GHz) could be achieved via transmission-line theory. Additionally, the corrosion current is reduced by two orders of magnitude in comparison to that of FCI and FCI@UF. Through in-depth analysis of the experimental results, the mechanism by which the UFC coating enhances electromagnetic wave attenuation and improves the weather resistance of FCI was explored.

## 2. Experimental Section

### 2.1. Raw Materials

Experimental Materials: Flake carbonyl iron powder (micron grade): analytical pure, from Changsha Liyou Metal Materials Co., Ltd., Changsha, China. The flake carbonyl iron powder needs to be ultrasonically cleaned with acetone and then dried in a vacuum oven for later use. Urea, formaldehyde (mass fraction 37%), hydrochloric acid (mass fraction 37%), ammonium chloride, n-octanol, acetone, anhydrous ethanol: analytical pure, from China National Pharmaceutical Group Chemical Reagent Co., Ltd., Shanghai, China. The hydrochloric acid with a mass fraction of 37% needs to be diluted with distilled water to prepare a 1% mass fraction of dilute hydrochloric acid for later use. Resorcinol: analytical pure, from Shanghai Macklin Industrial Park, Shanghai, China. OP-10: from Tianjin Zhixuan Chemical Reagent Co., Ltd., Tianjin, China. Distilled water: analytical pure, from Dayou Chemical Raw Materials Co., Ltd., Guangan, China.

### 2.2. Synthesis of FCI@UFC

**Figure 1:**
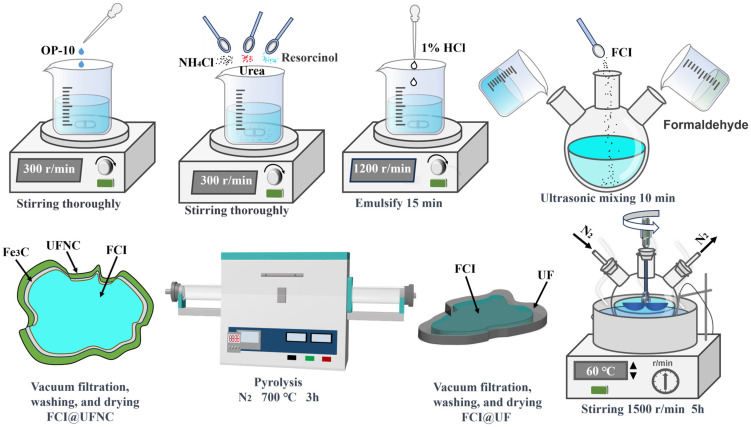
Synthesis process of FCI@UFC.

An amount of 2 g of OP-10 was introduced dropwise to 200 mL distilled water and stirred until fully dissolved at ambient temperatures. Subsequently, 18 g urea, 1.8 g ammonium chloride, and 1.8 g resorcinol were added to the above solution. The pH of the solution was adjusted to around 3 by slow addition of a 1% dilute hydrochloric acid solution, and it was emulsified at 1200 r/min for 15 min. The prepared emulsion was then transferred to a four-neck flask, followed by 20 g pre-treated FCI and 36 g formaldehyde solution, and stirred continuously in an oil bath at 60 °C for 5 h at 1500 rpm. Afterward, the FCI@UF precursor was obtained by vacuum filtration and a washing and drying process. The obtained precursor FCI@UF was thermally decomposed at 650 °C under a flowing nitrogen atmosphere for 3 h to obtain nitrogen-doped porous carbon-coated flake carbonyl iron composite (FCI@UFC), which was washed in anhydrous ethanol several times and then dried in a vacuum at 60 °C for 24 h before being utilized. The complete synthesis process for FCI@UF is shown in Figure 1.

### 2.3. Coating Preparation

The substrate material selected for this study was the 7B05 aluminum alloy, known for its high strength, good machinability, and excellent corrosion resistance [17,18]. The specifications of the alloy were 20 mm × 20 mm × 3 mm. The aluminum alloy was polished using 150-, 400-, and 600-grit sandpaper sequentially and then subjected to ultrasonic cleaning for 30 min. QH-15 anti-corrosive epoxy primer (Purchased from Ocean Chemical Research Institute Co., Ltd., Zigong, China) (components A and B mixed in a 1:1 volume ratio) was used as the coating binder. Subsequently, the absorber was added to the epoxy primer, with the absorber content accounting for 70% of the total coating mass. The mixture was evenly sprayed onto the aluminum plate using a spray gun, with multiple coats applied to ensure uniform coating thickness. After painting, the prepared aluminum alloy/coating electrodes were cured at room temperature in the atmosphere for 24 h.

### 2.4. Characterization Methods

**Figure 2:**
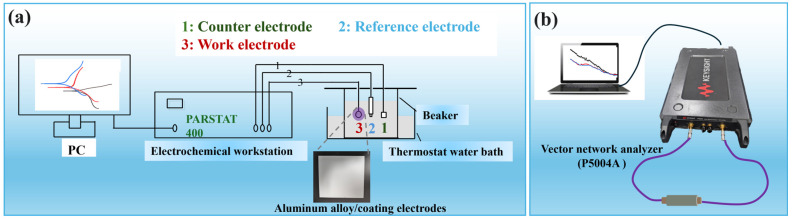
Schematic diagram of performance test. (**a**) Electrochemical performance; (**b**) electromagnetic response property.

A scanning electron microscope (SEM) equipped with an EDS attachment module was used to observe the microscopic morphology of the samples while analyzing their surface elemental composition and distribution. The crystal structure of the samples was verified using an X-ray diffractometer (XRD) with Cu target Kα radiation, a tube voltage of 40 kV, and a current of 40 mA. The electromagnetic response property was measured by a vector network analyzer (VAN, P5004A, Keysight Co., Ltd., Santa Rosa, CA, USA) through coaxial ring testing (Figure 2a). All samples were uniformly mixed with paraffin at a weight percentage of 70% and then processed to a toroidal shape with an outer diameter of 7 mm and inner diameter of 3.04 mm. Testing was conducted within the frequency range of 2~18 GHz, and electromagnetic parameters were simulated and calculated for reflection loss (RL) via transmit-line theory [19,20,21]. Electrochemical tests were conducted using a CS350 potentiostat, utilizing a three-electrode system (Figure 2b). The setup included an Ag/AgCl electrode as the reference electrode, a carbon rod as the counter electrode, and a coated aluminum alloy as the working electrode. Electrochemical Impedance Spectroscopy (EIS) was performed over a frequency range of 105 to 10^−2^ Hz with an AC voltage amplitude of 10 mV. Polarization curves were recorded using linear sweep voltammetry at a scan rate of 0.5 mV/s.

## 3. Results and Discussion

### 3.1. Phase Analysis

**Figure 3:**
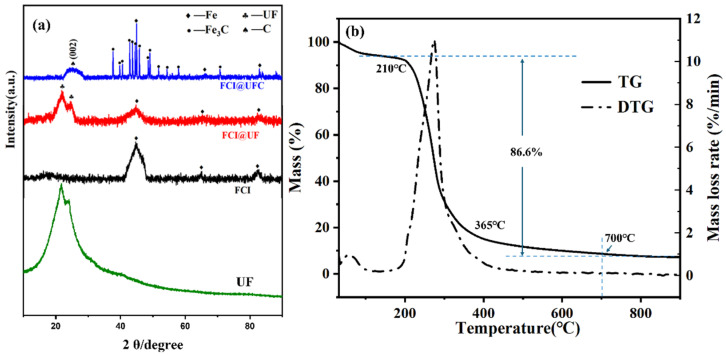
(**a**) XRD patterns of FCI, FCI@UF, UF@UFC; (**b**) The TG/DTG curves of pure UF in N2 atmosphere (room temperature—900 °C).

As shown in Figure 3a, it is clearly observed that the diffraction peaks at 44.69°, 65.05°, and 82.53° can be attributed to the (110), (200), and (211) crystal planes of the cubic system α-Fe (JCPDS No. 87-0721) [22]. For FCI@UF, in addition to the diffraction peaks of Fe, diffraction peaks occur at 2θ = 21.65° and 24.13°, which originate from the lattice planes of the cured resin. The above results are consistent with the reported literature [23], indicating the successful synthesis of the FCI@UF precursor. In the XRD spectrum of FCI@UFC, diffraction peaks of Fe are still present. Additionally, there is a relatively broad and low-intensity diffraction peak centered at 25.04° in the XRD pattern, corresponding to the (002) crystal plane of disordered carbon layer formed after carbonization (JCPDS No. 87-0721). Notably, the absence of the peak at 21.65° in the spectrum of FCI@UFC provides evidence for the complete transformation of UF into UFC. Moreover, the extra peaks match well with the Fe_3_C substance (JCPDS No. 35-0772), which is formed as a result of the reaction between Fe and C during carbonization [24]. The formation of Fe_3_C enhances the interfacial of the FCI@UFC and is expected to introduce additional loss mechanisms. In conclusion, the successful preparation of FCI@UFNC composite materials was preliminarily confirmed based on the analysis of the XRD pattern.

To further determine the carbonization temperature of the FCI@UF precursor, thermal analysis (TG/DTG) measurements in an N_2_ atmosphere were carried out on pure UF, as shown in Figure 3b. The TG curve exhibits a significant mass loss below 210 °C accompanied with an endothermic peak in DTG curve, which is believed to be from the volatilization of moisture on the material surface and residual monomers during the UF synthesis process. In the TG curve, significant mass loss (85.6%) occurs between 210–700 °C, corresponding to the thermal decomposition stage of UF. At this stage, the cross-linking points where the urea groups (-NH_2_) are located are destroyed, and the methylene groups in the main chain break, accompanied by the release of a large number of gases (ammonia (NH_3_), carbon dioxide (CO_2_), carbon monoxide (CO)), ultimately leading to structural changes and degradation of the polymer. This corresponds to the exothermic peak of 275 °C in the DTG curve. Based on the above analysis results, 650 °C is selected as the carbonization temperature for this work to completely carbonize FCI@UF in N_2_.

### 3.2. Powder Morphology and Microstructure

**Figure 4:**
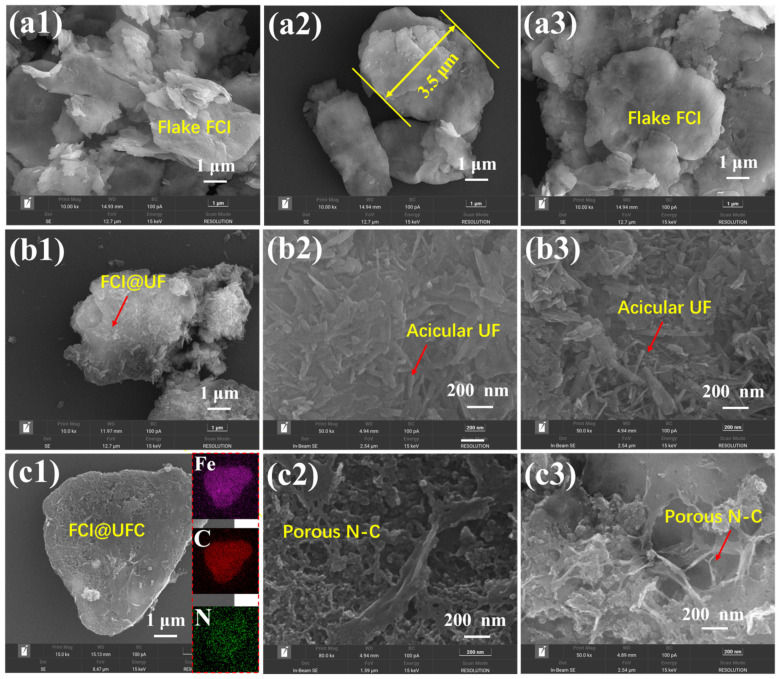
SEM Images: (**a1**–**a3**) FCI, (**b1**–**b3**) FCI@UF, (**c1**–**c3**) UF@UFC; the illustrations of (**c1**) are EDS mapping of Fe, C, and N elements.

The SEM micrographs of the samples are given in Figure 4. As observed in Figure 4a1–a3, the raw FCI displays a micrometer-sized (~3.5 μm) and flaky structure with a smooth surface. Figure 4b1 shows the SEM image of FCI@UF, and it can be observed that FCI@UF exhibits a similar morphology to that of the original FCI (Figure 4a2) but with a roughened surface. The high-resolution images (Figure 4b2,b3) reveal the formation of UF on the surface of FCI@UF, which is inferred to be a UF based on the XRD results (Figure 3a) [25]. After carbonization, it is evident that the surface morphology of the material undergoes a notable transformation, from needle-like to a distinctly porous structure (Figure 4c1–c3). This is attributed to the thermal decomposition reaction of the UF resin coating layer at high temperatures, resulting in the formation of a carbon layer and the release of gases such as carbon monoxide, nitrogen, and ammonia [26]. Existence of C and N elements on the surface of FCI@UFC composite is confirmed by EDS mapping in the inset of Figure 4c1, exhibiting distinct concentration distributions that provide further evidence for the conversion of UF into a carbon layer. As one knows, the above porous structure can provide an increased number of transmission paths for electromagnetic waves, thereby enhancing the reflection and scattering effects and ultimately improving the material’s electromagnetic wave absorption performance [27]. The presence of N atoms increases the electron cloud density of the material to some extent, which can improve the material’s conductivity.

### 3.3. Microwave Absorption Performance

#### 3.3.1. Electromagnetic Response Characteristics

**Figure 5:**
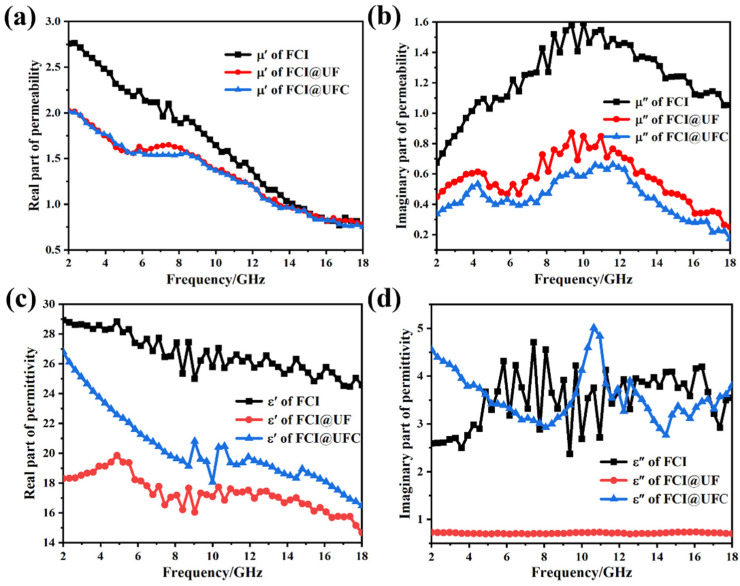
Variation of electromagnetic parameters with frequency for FCI, FCI@UF, and FCI@UFNC: (**a**) real parts of complex permeability (*μ*′), (**b**) imaginary parts of complex permeability (*μ*″), (**c**) real and parts of complex permittivity (*ε*′), and (**d**) parts of complex permittivity (*ε*″).

**Figure 6:**
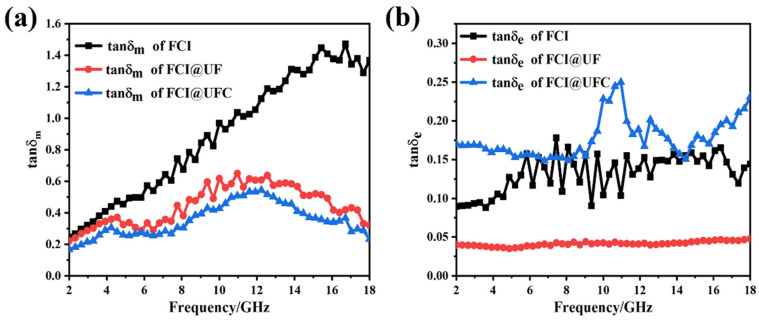
Loss tangents of FCI, FCI@UF, and FCI@UFNC: (**a**) magnetic loss tangent (tan*δ*_m_); (**b**) dielectric loss tangent (tan*δ*_e_).

Figure 5a,b, respectively, present the variations of the real part (μ′) and imaginary part (μ″) of FCI, FCI@UF, and FCI@UFC with frequency. In the graphs, as compared to FCI, both FCI@UF and FCI@UFC exhibit a decreasing trend in μ′ and μ″. This can be attributed to the non-magnetic UF or nitrogen-doped porous carbon situated between the magnetic FCI particles, which weakens the magnetic moment interactions among FCIs. As a result, the free rotation or flipping of magnetic moments is inhibited, leading to a decrease in the saturation magnetization of iron powder, thereby causing a reduction in the μ′ [28]. The presence of the coating layer also changes the coupling strength between electrons and magnetic moments. This alteration leads to a reduction in the energy transfer under external alternating magnetic fields and a weakening of the interaction between magnetic moments and these external alternating magnetic fields. Hence, the imaginary part of the magnetic permeability decreases [29]. As a result, both FCI@UF and FCI@UFC experience a decline in μ′ and μ″. In Figure 6a, FCI@UFC exhibits the lowest tanδm, which indicates the weakest magnetic loss performance among them.

Figure 5c,d illustrate the variations of the real parts (ε′) and imaginary parts (ε″) of the complex permittivity with frequency for the three composite materials. Due to the electrical insulating nature of UF, the movement of electrons is hindered after UF coating, which affects the conductivity and polarization effects of the iron powder. This significantly decreases the ε′ and ε″ of FCI@UF compared to the original FCI [30]. After high-temperature carbonization of FCI@UF, the surface-nitrogen-doped porous carbon material enhances the electron mobility and conductivity of FCI. This improvement can be explained through the theory of free electrons [31]:

(1) $$\varepsilon^{\prime \prime }\approx \sigma /2\pi \varepsilon_{0}f$$

In the equation, σ represents the electrical conductivity, ε_0_ is the dielectric constant in vacuum, and f denotes the frequency. According to the formula, ε″ is directly proportional to σ. Therefore, the ε″ of FCI@UFC increases. Furthermore, from Figure 4c1–c3 it can be observed that the ε′ of FCI@UFC is smaller than that of FCI. This is due to the fact that the increased electron mobility leads to more effective electron participation in polarization under an external electric field, enhancing ε″. Simultaneously, it also leads to a stronger separation of electrons under the electric field, which weakens the polarization response of molecules or atoms in the material, thereby causing a decrease in the ε′ of FCI [32,33]. In Figure 6b, FCI@UFC exhibits the highest tanδ_e_, which indicates that nitrogen-doped porous carbon enhances the dielectric loss capability of FCI. The presence of the carbon layer is utilized to modify the dielectric properties of FCI, thereby improving its absorption performance.

#### 3.3.2. Electromagnetic Wave Absorption Characteristics

FCI@UFC composite material primarily enhances its absorption performance by optimizing the electrical conduction loss and polarization loss. Electrical conduction loss is caused by the migration of free electrons within the material, and the introduction of nitrogen-doped porous carbon layers is beneficial for the formation of an electron network. This allows electrons to move more easily through mechanisms such as migration, hopping, and tunneling within the conductive channels, generating induced currents. These induced currents convert electromagnetic wave energy into thermal energy, dissipating it [34]. Additionally, the inherently high electron mobility of carbon-based materials facilitates the rapid transfer of electrons, enhancing energy conversion efficiency and increasing electrical conduction loss [35]. Under the influence of an external electromagnetic field, the charge distribution and orientation of electrons, ions, or molecules within the material change, forming dipoles. When the external field frequency changes, the electric dipoles in the material rapidly adjust according to the external field frequency, leading to friction and collisions within and between molecules, resulting in the dissipation of electromagnetic wave energy. In FCI@UFC, electrons can undergo multi-level charge transfer between FCI, Fe_3_C, and the carbon layer, causing charge separation within molecules and increasing the number of dipoles [36].

**Figure 7:**
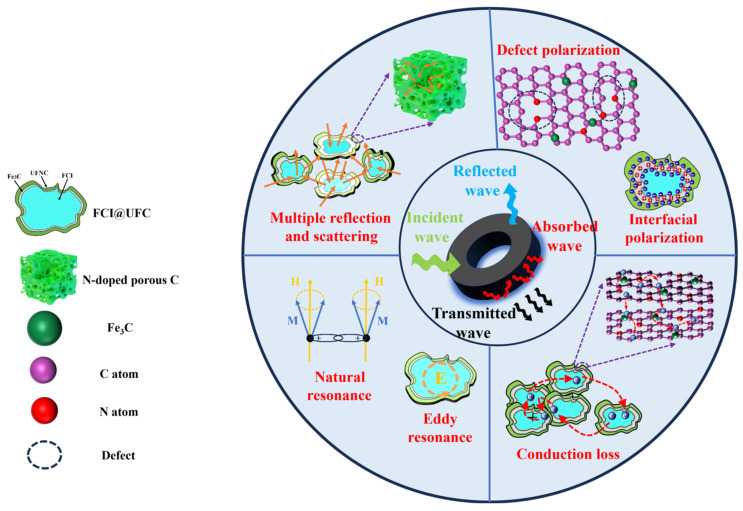
Schematic illustration of microwave absorption mechanism for FCI@UFC composite material.

Furthermore, heterogeneous interface structures are formed between FCI, Fe3C, and the carbon layer, capturing a large number of electrons and ions at the interface to form dipoles [36]. Defects caused by in situ doping of N atoms also act as dipoles. The increase in the number of electric dipoles within FCI@UFC implies more frequent relative displacements and energy dissipation phenomena, consequently resulting in greater dielectric loss [37]. The absorption mechanism of FCI@UFC is illustrated in Figure 7. Compared to FCI, FCI@UFC exhibits reduced magnetic loss and enhanced dielectric loss of electromagnetic wave energy. To directly assess the material’s absorption performance strength, the reflection loss of the metal-substrate single-layer wave-absorbing coating is simulated by measured electromagnetic parameters based on the transmission line theory [38]:

(2) $$\mathrm{RL}=20\mathrm{lg}\left|Z_{\mathrm{in}}-Z_{0}/Z_{\mathrm{in}}+Z_{0}\right|$$

(3) $$Z_{0}=\sqrt{{\mu_{0}/\varepsilon }_{0}}$$

(4) $$Z_{\mathrm{in}}=\sqrt{\mu_{0}\mu_{r}/\varepsilon_{0}\varepsilon_{r}}=Z_{0}\sqrt{\mu_{r}/\varepsilon_{r}}\tanh\left\{j\left(2\pi \mathrm{fd}/c\right)\sqrt{\mu_{r}\varepsilon_{r}}\right\}$$

In the equation, Z_0_ represents the characteristic impedance of air, Z_in_ is the input impedance of the absorptive material, μ_0_ and ε_0_, respectively, denote the magnetic permeability and dielectric constant of air. *μ*_0_ = 4π × 10^−7^ H·m^−1^, *ε*_0_ = 8.85 × 10^−12^ C^2^·N^−1^ m^−2^. The formula yields Z_0_ = 377 Ω. z represents the thickness of the absorber, c is the speed of light, *μ*_r_ = *μ*′ − j*μ*″ represents the relative magnetic permeability of the absorptive material, and *ε*_r_ = *ε*′ − j*ε*″ represents the relative dielectric constant of the absorptive material.

**Figure 8:**
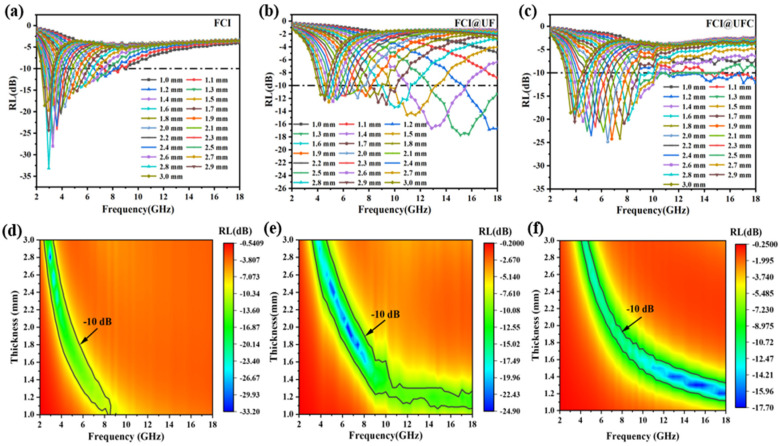
Two-dimensional RL surface plots and corresponding contour maps: (**a**,**d**) FCI, (**b**,**e**) FCI@UF, (**c**,**f**) UF@UFC.

Figure 8 displays three-dimensional RL surface plots and corresponding contour maps for FCI, FCI@UF, and FCI@UFC absorbers with thicknesses ranging from 1 to 3 mm within the frequency range of 2–18 GHz. In Figure 8a–c, within the thickness range of 1–3 mm, FCI exhibits RL values below −10 dB between 2.35–8.54 GHz, with the effective absorption band concentrated in the lower-frequency region. This phenomenon can be attributed to the rapid change in electromagnetic field at higher frequencies. Additionally, the relatively high conductivity of FCI results in frequent eddy currents circulating within the conductor. Consequently, the magnetic field in FCI is predominantly confined near the surface, with minimal magnetic field penetration into its interior. This leads to a substantial portion of electromagnetic waves being reflected back to the surrounding air environment, a phenomenon known as the eddy current effect [39]. In Figure 8b–e, the introduction of the UF coating layer enhances the absorptive performance of FCI. The effective absorption band becomes broader and shifts towards higher frequencies. Specifically, at a thickness of z = 1.3 mm, the frequency range with RL < −10 dB is between 15.44 and 18 GHz. This improvement is attributed to the UF coating layer, which increases the overall electrical resistivity of the material, thereby reducing the adverse effects of the skin effect caused by eddy currents. Consequently, more electromagnetic waves penetrate the absorber material’s interior and are absorbed [40]. As shown in Figure 8c–f, compared to FCI and FCI@UF, FCI@UFC achieves a wider effective absorption band with a smaller thickness. FCI@UFC effectively combines the magnetic loss of FCI with the dielectric loss of the carbon layer, exhibiting dual-loss characteristics [41]. The nitrogen-doped porous carbon layer mitigates direct contact and magnetic coupling between magnetic particles at the physical level. Meanwhile, its porous structure with inherent defects enhances electromagnetic wave scattering at the microscopic level, thereby strengthening wave–material interaction and promoting charge carrier migration and scattering within the composite material. Furthermore, at the chemical level, the charge carriers and defects introduced by nitrogen doping act as additional polarization centers, influencing the carrier migration and recombination processes, thereby leading to higher dielectric loss at high frequencies. The nitrogen-doped porous carbon layer functions at both the physical and chemical levels, enabling the composite material to exhibit more complex electromagnetic wave interactions at high frequencies, including enhanced absorption and altered scattering mechanisms. This significantly enhances the microwave absorption efficiency of the FCI@UFC composite material at high frequencies and also explains why the peaks of complex permeability and complex permittivity occur at different frequencies compared to the maximum reflectivity loss.

**Figure 9:**
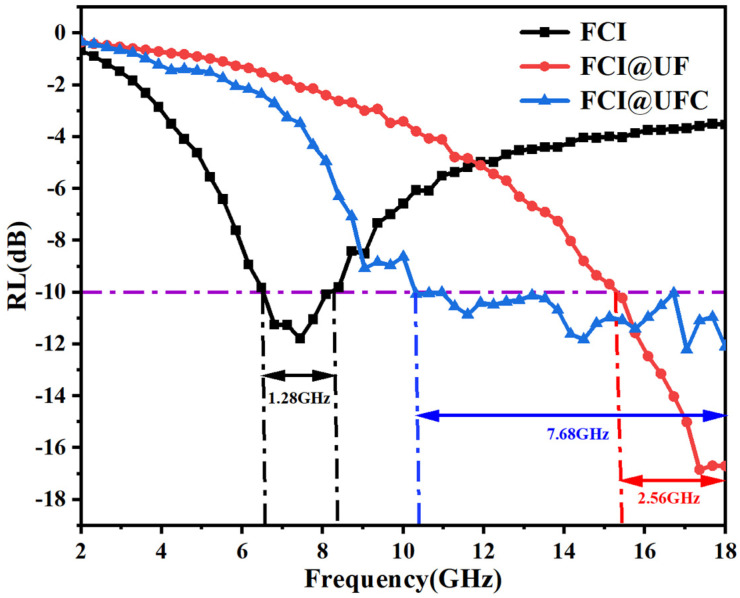
Comparison of reflectivity loss (RL) for FCI, FCI@UF, and FCI@UFC composite materials (thickness 1.2 mm).

When simulating an absorption layer thickness of 1.2 mm, the effective absorption bandwidth with RL < −10 dB ranges from 10.32 to 18 GHz. Figure 9 displays the RL curves of the three samples at z = 1.2 mm, providing a clearer presentation of the differences in the microwave absorption performance between FCI, FCI@UF, and FCI@UFC.

Table 1 compares our research results with the recent outstanding achievements published on FCI-based composite materials. FCI@UFC has a higher EAB value and a lower filling ratio. In practical applications, it is expected to meet the requirements of electromagnetic wave absorbers that are lighter, thinner, and stronger and have a wider bandwidth.

### 3.4. Corrosion Resistance

In order to evaluate the corrosion resistance of FCI@UFC, absorption coatings were prepared by separately mixing FCI, FCI@UF, and FCI@UFC with epoxy primer. The corrosion characteristics of the three coatings in a NaCl neutral solution were analyzed through electrochemical testing.

**Figure 10:**
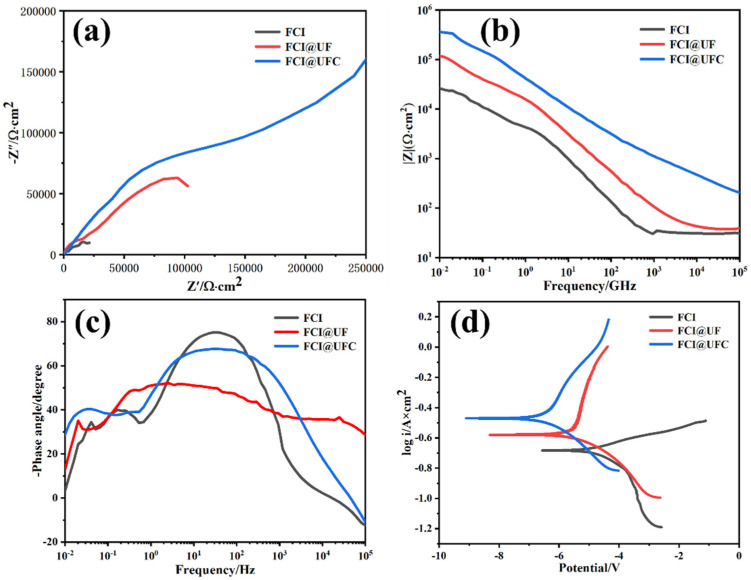
Electrochemical curves for FCI, FCI@UF, and FCI@UFC: (**a**) Nyquist plots, Bode plots: (**b**) relationship between impedance modulus and frequency, (**c**) relationship between phase angle and frequency, (**d**) Tafel plots.

Figure 10a displays the Nyquist plots of the three coatings, where the capacitive arc radius is directly proportional to the corrosion resistance of the coatings. A larger capacitive arc radius indicates better corrosion resistance of the coating [45]. The capacitance arc radius showed FCI@UFC > FCI@UF > FCI, indicating that FCI@UFC has better corrosion resistance. This is because FCI@UFC combines the advantages of encapsulation and porous structure [46]. A carbon coating layer with good chemical stability can act as a physical barrier. It reduces direct contact between corrosive ions and FCI, thereby enhancing the overall corrosion resistance of FCI@UFC [25]. On the other hand, the carbon coating layer with numerous small pores and cavities can provide more active sites, aiding in electron conduction and ion transport. The porous structure can also accommodate electrolytes within the coating, thereby maintaining a stable electrochemical environment and promoting improved corrosion resistance [47].

Figure 10b,c present the Bode plots of the three coatings, where corrosion reactions typically occur in the low-frequency region. The impedance modulus and phase angle at low frequencies (0.01 Hz) are crucial indicators for evaluating the corrosion resistance performance of the materials [48]. FCI@UFC exhibits the highest impedance modulus and phase angle in the low-frequency region. This observation aligns with the analysis from the Nyquist plots, thereby providing confirmation of its excellent corrosion resistance in a neutral NaCl solution.

Figure 10d presents the potentiodynamic polarization curves of the three coating samples in a 3.5 wt% NaCl solution at room temperature. Anodic Tafel slope (*β*_a_), cathodic Tafel slope (*β*_c_), corrosion potential (*E*_corr_), corrosion current (*I*_corr_), corrosion rate (CR), and polarization resistance (*R*_p_) were obtained through the Tafel extrapolation method. The calculation formulas for CR and *R*_p_ are provided below. The results are listed in Table 2 [49,50]:

(5) $$\mathrm{CR}=\frac{I_{\mathrm{corr}}\times K\times \mathrm{EW}}{\rho A}$$

(6) $$R_{P}=\frac{\beta_{a}\beta_{c}}{I_{\mathrm{corr}}In(10)(\beta_{a}+\beta_{c})}$$

In the formula, K represents the corrosion rate constant for iron, with K ≈ 3272 mm/year (1.038 × 10^−7^ m/s); EW denotes equivalent weight, which in this paper refers to the equivalent weight of iron (27.92 g/equivalent); and ρ is the density of iron (7.86 g/cm^2^). As evident from Figure 10d and Table 2, compared to pure FCI, the *E*_corr_ of FCI@UFC coating shifted in the positive direction, increasing from −0.682 V to −0.472 V, while *I*_corr_ decreased from 3.25 × 10^−5^ A/cm^2^ to 1.45 × 10^−7^ A/cm^2^, dropping by two orders of magnitude. Simultaneously, from FCI to FCI@UFC, CR reduced significantly from 0.2651 mm/a to 0.0432 mm/a. *R*_p_ increased from 165 Ω to 5214 Ω, further confirming the superior corrosion resistance of FCI@UFC.

## 4. Conclusions

In this study, FCI@UFC composite particles were successfully synthesized through an in situ polymerization method followed by a subsequent pyrolysis process. While retaining the flake-like morphology of iron powder, the unique microstructure and phase composition were introduced to enhance the electrical conductivity and polarization loss of the original FCI, thereby realizing a magnetoelectric synergistic effect between the nitrogen-doped porous carbon coating layer and the iron powder. At higher frequencies, the dominant contribution to absorption is attributed to the dielectric loss of the carbon layer, while at lower frequencies, the magnetic loss of the iron powder remains effective, broadening the effective absorption bandwidth of the initial FCI. FCI@UFC, with a simulated absorption layer thickness of 1.2 mm, achieves an impressive absorption bandwidth of 7.68 GHz (10.32–18 GHz) and demonstrates outstanding microwave absorption performance. Furthermore, the nitrogen-doped porous carbon layer exhibits excellent chemical stability, acting as a barrier layer to hinder the contact between the corrosive medium and FCI. The porous structure of the carbon layer also aids in maintaining a stable electrochemical environment, thereby imparting superior corrosion resistance to the iron powder. Electrochemical test results indicate that compared to pure FCI, the corrosion current of the FCI@UFC composite material is reduced by two orders of magnitude, with the *R*_p_ increasing from 165 Ω to 5214 Ω, leading to a significant reduction in corrosion rate. In summary, the presence of the nitrogen-doped porous carbon layer enhances the microwave absorption performance and corrosion resistance of FCI. These findings provide a novel approach for expanding the application scope of FCI and developing composite materials with excellent microwave absorption and corrosion resistance.

## Figures and Tables

**Table 1:** Comparison of EMM absorption performance between FCI@UFC developed in this study and FCI-based composites reported in other excellent recent studies.

Composites	RLmin (dB)	Thickness (mm)	EAB (−10 dB) (GHz)	Percentage (wt %)	Ref.
FCI@SiO_2_	−7	1.2	0	70	[14]
CI/CNT-50	−23	1.5	5.2	50–55	[42]
FCI/CFB(Fiber)	−13.5	1.2	4.5	65	[43]
FCI@Co/C	−72.6	1.5	6.2	60	[44]
FCI@UFC	−12	1.2	7.68	60	This work

**Table 2:** Tafel curve analysis results.

Sample	*β*_a_ (V/dec)	*β*_c_ (V/dec)	*I*_corr_ (A/cm^2^)	*E*_corr_ (V)	CR (mm/a)	*R*_p_ (Ω)
FCI	36.24	72.13	3.25 × 10^−5^	−0.682	0.2651	165
FCI@UF	231.45	69.5	2.31 × 10^−6^	−0.501	0.0945	2169
FCI@UFC	183.18	64.2	1.45 × 10^−7^	−0.472	0.0432	5214

## Data Availability

The original contributions presented in this study are included in the article. Further inquiries can be directed to the corresponding author.
